# Partial sternectomy with reconstruction of a giant cell tumor of the sternum, a case report, Saudi, Arabia

**DOI:** 10.1186/s13019-023-02404-0

**Published:** 2023-10-17

**Authors:** Safwat Eldaabossi, Yasser Al-Ghoneimy, Ahmad Antar, Elsaid Lotfy, Hameed Aljawad, Yasser G. Abish, Mohammed Helyl, Haytham Oraby, Hesham Soliman, Bassam Abdullatif, Sameh O. Nour, Ahmad Lotfi

**Affiliations:** 1 Almoosa Specialist Hospital, Al Ahsa, Saudi Arabia; 2 Department of Chest Diseases, Al-Azhar Faculty of Medicine, Cairo, Egypt; 3 Radiology Department, Almoosa Specialist Hospital, Al Ahsa, Saudi Arabia; 4 Pathology and Laboratory Medicine, Almoosa Specialist Hospital, Al Ahsa, Saudi Arabia; 5 Al-Azhar Faculty of Medicine, Cairo, Egypt; 6 Anesthesia Department, Almoosa Specialist Hospital, Al Ahsa, Saudi Arabia

**Keywords:** Giant cell tumors, Sternum, Subtotal sternectomy

## Abstract

**Background:**

Giant cell tumor (GCT) is a relatively common and locally aggressive benign bone tumor that rarely affects the sternum.

**Case presentation:**

We report a case of giant cell tumor of the sternum in a 28-year-old Saudi with painful swelling at the lower part of the sternum. Subtotal sternectomy and reconstruction with a neosternum using two layers of proline mesh, a methyl methacrylate prosthesis, and bilateral pectoralis muscle advancement flaps were performed.

**Conclusions:**

Giant cell tumor of the sternum is a rare diagnosis. Surgical resection with negative margins is the ideal management. To avoid defects or instability of the chest wall, reconstruction of the chest wall with neosternum should be considered.

## Background

Giant cell tumor (GCT) is a relatively common benign bone tumor that can be locally aggressive, although it rarely affects the sternum. Primary sternal tumors are very rare, with secondary sternal tumors being more common [1, 2]. GCT is a neoplasm of mesenchymal origin characterized by the proliferation of osteoclastic multinucleated giant cells on a background of mononuclear cell stroma. In rare cases, GCT may undergo malignant transformation and often recurs locally after surgery [1, 2]. GCTs account for 5–6% of all primary bone tumors, with an incidence of primary malignancy accounting for 1.6% of all GCTs. They usually occur in the meta-epiphyseal region of the long tubular bones, and involvement of the sternum by GCT is very rare, with only a few cases reported [1–4].

## Case presentation

**Fig. 1:**
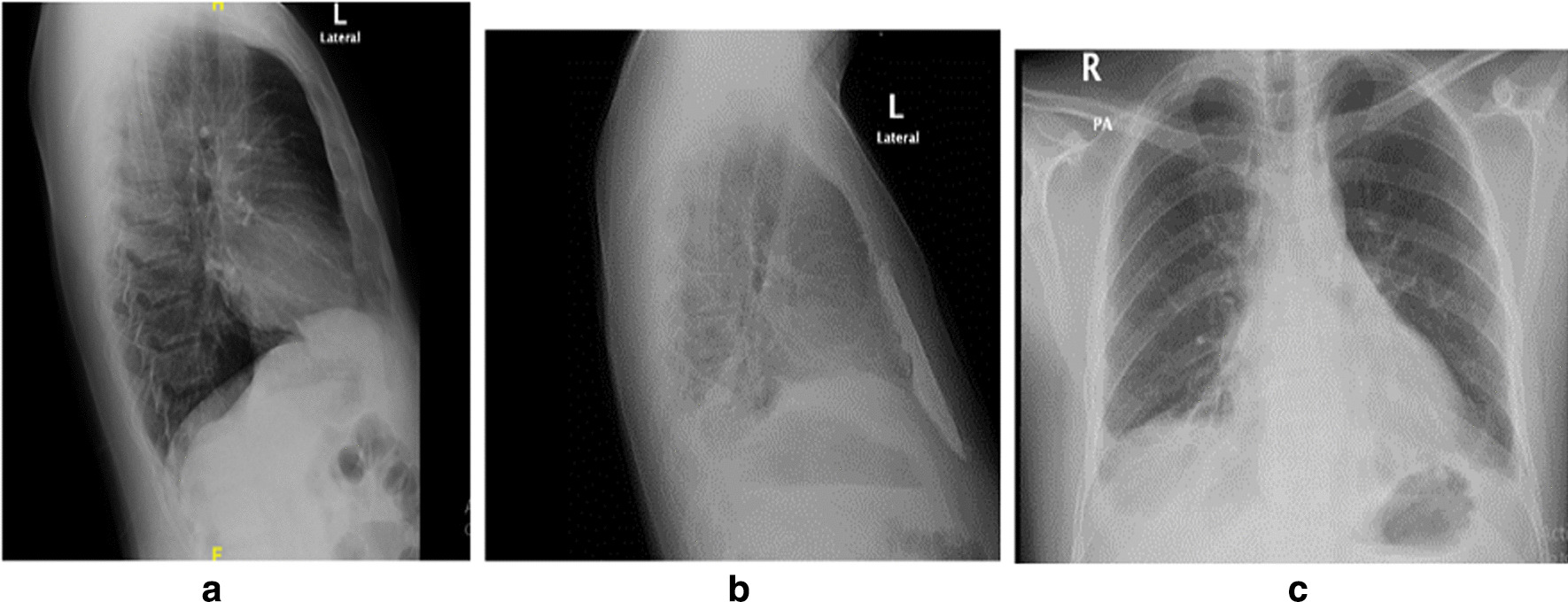
**a** Chest radiograph lateral view: showed swelling at the lower part of the sternal body. **b** Chest radiograph lateral view: showed synthetic material at the lower part of the sternal body. **c** Chest radiograph posteroanterior view: showed blunted both costophrenic angles

**Fig. 2:**
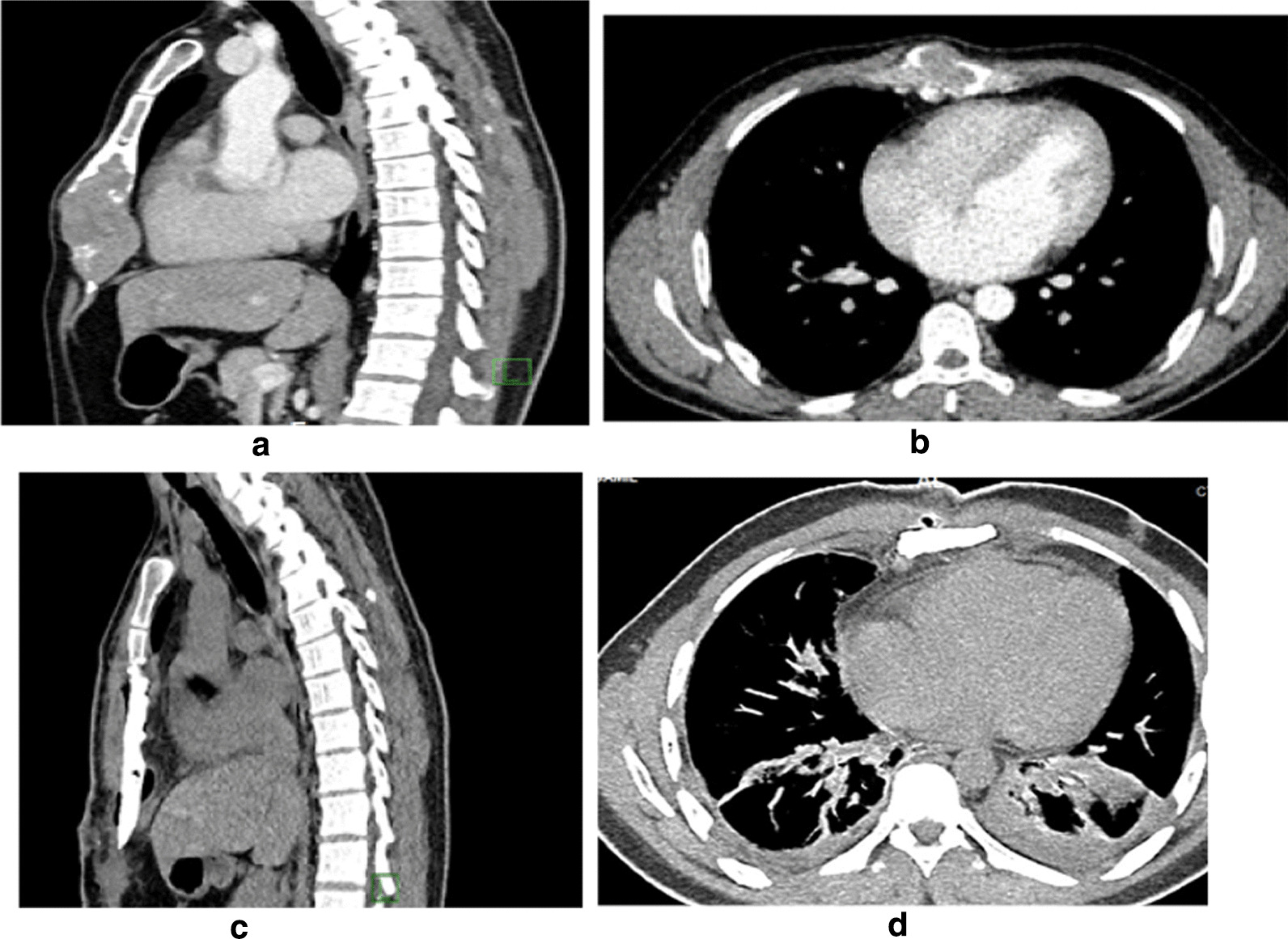
CT Chest mediastinal window: sagittal (**a**) and axial, (**b**) mediastinal window: showed large, expanding lytic lesion in the lower two-thirds of the sternum. CT Chest mediastinal window, **c** Sagittal midline sternum displayed post- operative after lower sternal tumor resection and reconstruction with replacement with prosthetic material. **d** Axial mediastinal window: bilateral mild pleural effusions with consolidation collapse of the lower lobes and subsegmental atelectasis. Air bubbles in the sternal surgical bed and subcutaneous edema without gross residual masses or localized fluid collections

On September 29, 2022, a 28-year-old nonsmoking, Grocery seller Saudi patient presented with a 3-month history of persistent dull pain and gradually increasing swelling in the lower sternum. The patient denied fever, cough, dyspnea, or chest trauma. Physical examination revealed a hard mass in the lower sternum, while chest radiography lateral view showed soft tissue lesion at the lower sternum (Fig. 1a). Computed tomography (CT) of the chest showed a large, expansile lytic lesion in the lower two-thirds of the sternum, likely representing an underlying bone tumor. Magnetic resonance imaging (MRI) of the chest showed a sternal large medullary lesion with a bony extent of 8.5 × 4 × 3.5 cm, which was mostly consistent with a benign chondroma or malignant chondrosarcoma (Fig. 2a, b).

Under general anesthesia, multiple True-Cut biopsies were obtained from the sternal expansile soft tissue mass using a 16-gauge needle. Histopathologic examination of the sternal biopsy revealed multiple cores and bone fragments, most of which were replaced by cellular lesion tissue consisting of numerous multinucleated giant cells seen as aggregates or scattered cells and mononuclear round to oval cells. The histologic conclusion is of giant cell-rich lesion with p63 immunohistochemistry nuclear positivity within the mononuclear cells which is in keeping with giant cell tumor of bone.

## Operative procedure

### Subtotal sternectomy with reconstruction

**Fig. 3:**
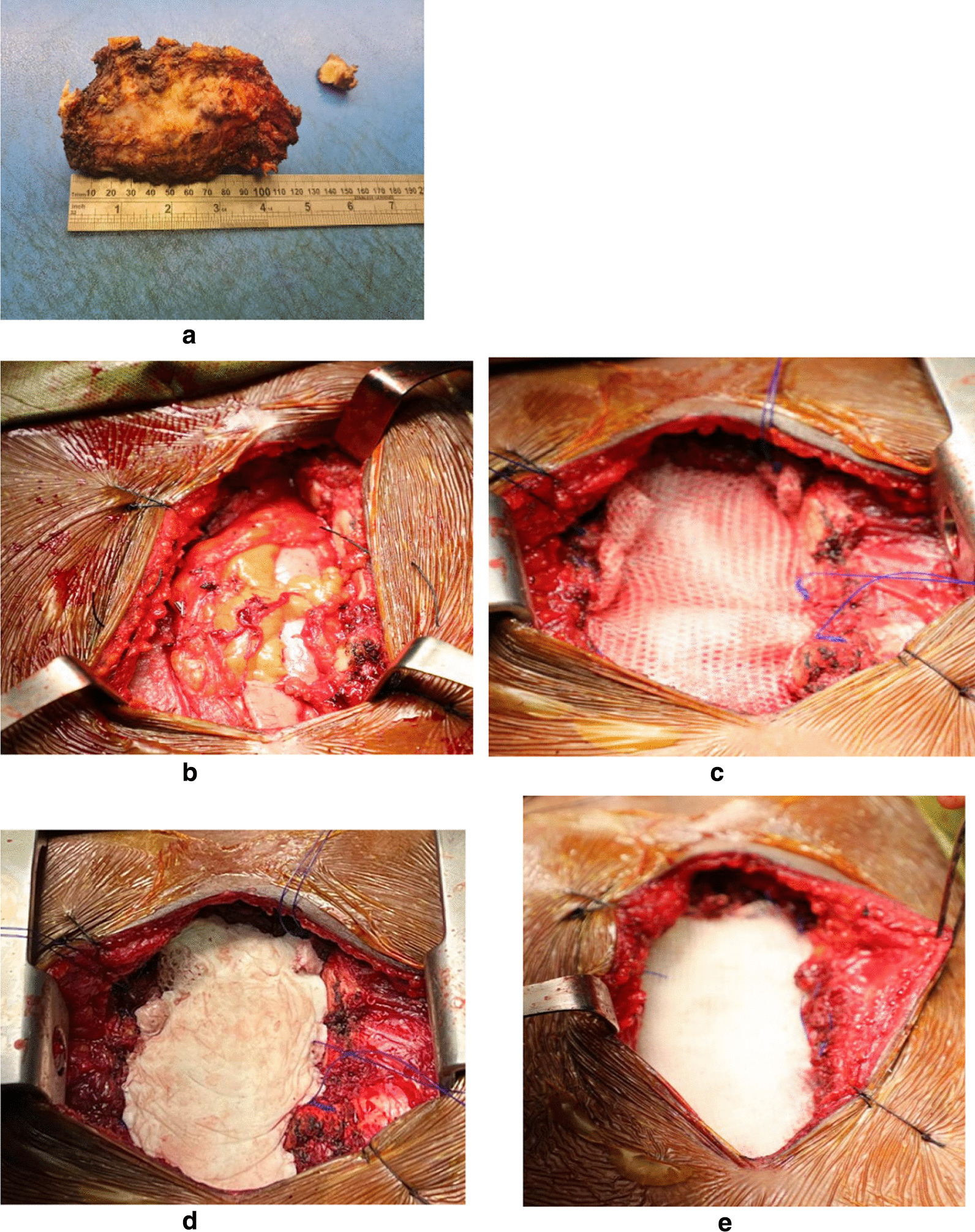
**a** The resection specimen of the sternal tumor with safety margin. **b** After subtotal resection of the sternum, the underlying pericardium and lung are unaffected. **c** The first layer of polypropylene mesh sutured to the surrounding tissue with monofilament nonabsorbable sutures. **d** The methyl methacrylate is applied to achieve stiffening of the chest wall. **e** The second layer of polypropylene mesh covering the methyl methacrylate cement

On December 29, 2022, the patient underwent excision of the sternal mass under general anesthesia (Fig. 3a), which was replaced by artificial bone. The operative procedure involved subtotal sternectomy with reconstruction. Under general anesthesia, the patient was placed in the supine position. The skin over the sternal mass was incised transversely, and the subcutaneous tissue was transected down to the sternal mass. The sternal resection included a wide resection of the affected portion of the sternum with a bone margin of 3 cm. The resection started above the rib margins, sparing the unaffected lateral portion of the pectoralis major muscle. A subtotal sternectomy was performed, omitting the uppermost 2 cm of the manubrium and clavicles (Fig. 3b). The left internal mammary artery was ligated, while the right was spared. The underlying pericardium and lung were not affected by the tumor. Thoracic drains were inserted before chest wall reconstruction.

### Sternal reconstruction

Chest wall stability was achieved with two layers of proline mesh. The first layer was sutured to the edges of the sternal defect with nonabsorbable monofilament nylon sutures (Fig. 3c). Subsequently, chest wall stability was reinforced with methyl methacrylate. The methyl methacrylate was spread on the mesh and anchored in the surrounding tissue (Fig. 3d). A second layer of proline mesh was then sutured to the surrounding sutures covering the methyl methacrylate (Fig. 3e). Soft tissue coverage of the sternal defect was provided by the pectoralis major muscles with skin advancement. The patient was stably extubated. The postoperative course was complicated by bilateral lower lobe collapse (Fig. 2c, d), which was treated with a systemic broad-spectrum antibiotic, and was discharged in good condition.

**Fig. 4:**
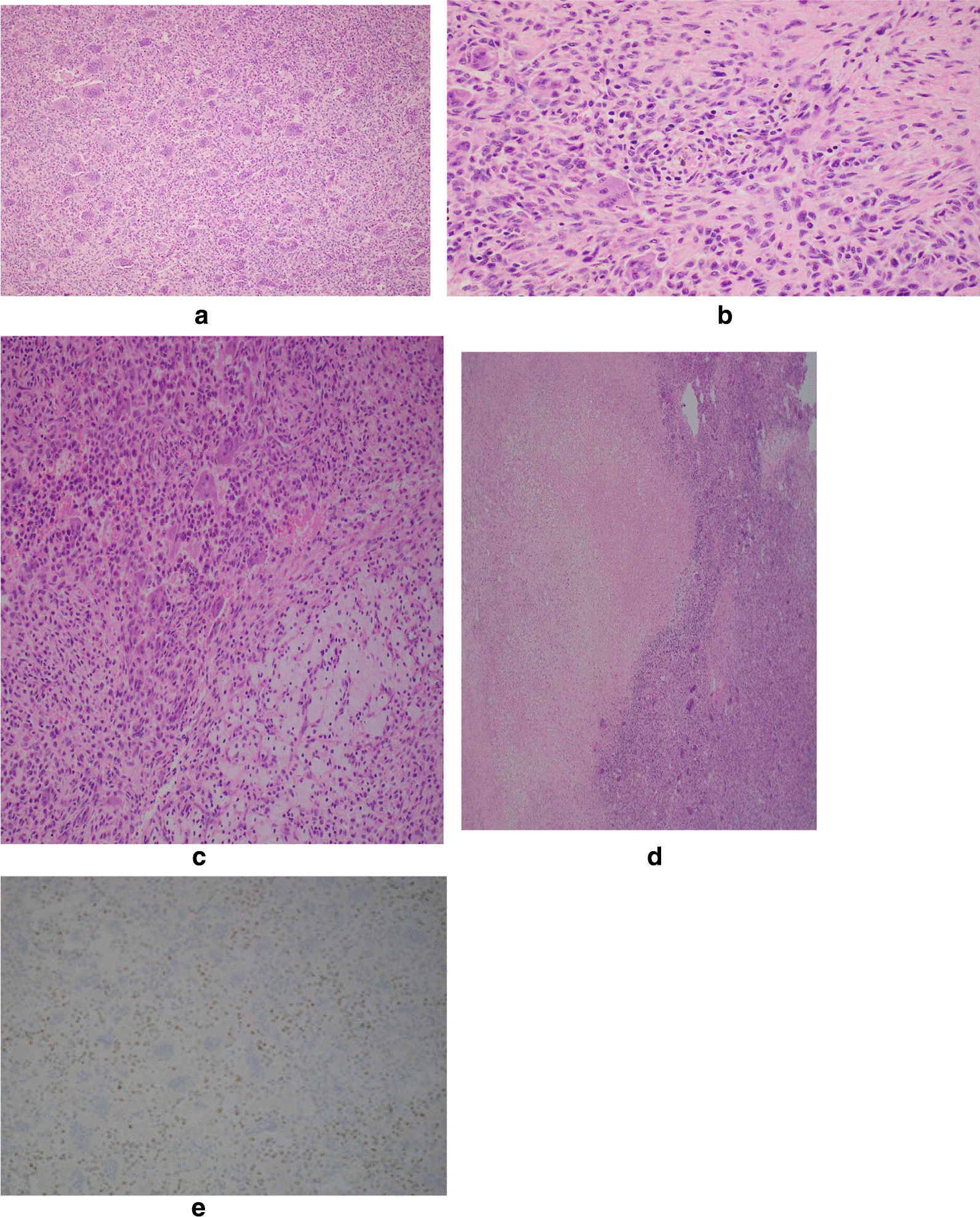
**a** Cellular lesion comprised of multinucleated giant cells containing numerous nuclei and monomorphic mononuclear cells. The stroma contains minimal if any collagen. **b** Hemosiderin deposits within stroma and histocytes. **c** Aggregate of foamy histiocytes. **d** Tumor necrosis with ghost of mononuclear tumor cells admixed with fibrin and histiocytes. **e** p63 immunohistochemistry highlighted the nuclei of the mononuclear cells which supports the diagnosis of giant cell tumor

Histopathological examination of the resected sternal specimen showed a cellular lesion composed of multinucleated giant cells and monomorphic mononuclear cells. The multinucleated giant cells were observed in aggregates or as single scattered cells, with numerous nuclei, some containing more than 30 nuclei per cell (Fig. 4a). The monomorphic mononuclear cells were polygonal, round, or spindly, without cytologic atypia. These cells were primarily embedded in a cellular mononuclear stroma with minimal collagen, although focal areas showed collagen and cartilage, and hemosiderin deposits were found within the stroma and histiocytes (Fig. 4b). In some areas, aggregates of foamy histiocytes were present (Fig. 4c). Mitotic figures were scattered within the mononuclear cells but not within the giant cells. Tumor necrosis was also observed in some areas (Fig. 4d). No osteoid formation was noted. Immunohistochemical staining for p63 was repeated in the tissue of the resected specimen and showed diffuse nuclear positivity in the mononuclear cells (Fig. 4e). Our case did not have the histologic features of a nonossifying fibroma: no spindle-shaped fibroblasts arranged in a storiform pattern. There was no characteristic round or polyhedral chondroblasts with abundant eosinophilic cytoplasm and well-defined cell borders with a chondroid matrix suggestive of chondroblastoma. Histologic examination of the removed tissue revealed no evidence of a preexisting aneurysmal bone cyst with secondary giant cell tumor. In addition, our case did not have the high-grade morphologic atypia characteristic of giant cell-rich osteosarcoma. Furthermore, the presence of p63 positivity in the mononuclear cells in our case was not consistent with nonossifying fibromas, chondroblastoma, or osteosarcoma tumor cells.

After 2-week, regular follow up in the cardiothoracic surgery clinic was done, he was asymptomatic and follow up chest radiograph synthetic material at the lower sternum (Fig. 1b, c).

## Discussion

Giant cell tumors (GCTs) of the bone are locally aggressive neoplasms that are rarely malignant or metastatic. They commonly occur at the end of long bones and can be classified as either conventional or malignant. Secondary malignant transformation after treatment is more common than primary malignant variants [4, 5]. Sternal GCTs are extremely rare and can be life-threatening due to their metastatic potential and possible mediastinal compression, leading to sudden death [6]. Only 13 cases of sternal GCT have been reported, with the sternal body involved in 61.5% and the manubrium in 38.4%. The mean age was 45 years (with a range of 28–74 years), and most cases were men (66.4%) [1].

The main clinical manifestation is intermittent localized pain with or without swelling in the affected region. The pain may be caused by local invasion of the thin cortical layer of the sternum. Signs of inflammation have been noted, especially in the sternoclavicular areas [7]. Our case is 28-year man, presented with sternal pain for 3 months.

Chest radiography is of limited value in diagnosing small sternal lesions and evaluating intrathoracic extension; lateral radiographs may be helpful, especially in distinguishing intra- and extrathoracic lesions. CT is the preferred method for evaluation and provides excellent anatomic detail to the surgeon preoperatively, especially in determining intrathoracic extension and mediastinal invasion. MRI is indicated to define the characteristics of GCT as a solid cystic lesion [6]. In our case lateral chest radiograph detected lower sternal lesion retrograde and the diagnosis of sternal mass was done by CT and MRI.

For accurate morphologic diagnosis of GCT in chest wall tumors, incisional biopsy is usually required [8]. Sternal biopsies are also technically challenging, with a potential for puncture of the aortic arch, pleural space, and lungs. CT-guided percutaneous sternal biopsy has a relatively lower diagnostic yield [9]. Our case was diagnosed by CT guided true cut needle biopsy under GA and the diagnosis was finalized after tumor resection as GCT.

Histologic examination of the tumor reveals two major components: non-neoplastic multinucleated giant cells and neoplastic mononuclear cells that are round to oval to spindle-shaped. The giant cells and tumor cells are distributed without a specific or characteristic pattern, and the background stroma typically contains a small amount of collagen. The tumor and stroma may also contain variable amounts of hemosiderin, macrophages, necrosis, and hemorrhage [10, 11]. In difficult cases, immunohistochemistry for p63 [12] and H3G34W [13] can be used to support the diagnosis of GCT because these immunohistochemical stains are considered specific for GCT in the appropriate clinical and histologic setting. In addition, the presence of p63 positivity in the mononuclear cells in our case excluded nonossifying fibroma, chondroblastoma, and osteosarcoma.

The classic treatment for GCT of bone is curettage, usually in combination with local adjuvants, but local recurrence is common within 2 years. En-bloc resection is associated with a lower recurrence rate but higher morbidity [4]. Subtotal sternectomy is the most common procedure for GCT of the sternum, and the extent of resection depends on the location of the tumor [1, 2, 8]. To avoid pulmonary complications such as a flutter chest and paradoxical breathing, the sternum should be adequately reconstructed after tumor resection. This will also protect the underlying structures and result in better function and cosmetic outcome [1–3]. Of the 13 reported cases of sternal GCT, the sternal body was involved in 9 cases (69.2%), and subtotal sternectomy was performed in 8 of 9 cases (88.8%) [1]. Futani et al., suggest that prosthetic replacement is required after subtotal sternectomy to protect the lungs, heart, and major vessels and to restore functional thoracic motion to prevent paradoxical breathing [14]. Our case underwent excision of the sternal mass under general anesthesia, which was replaced by artificial bone.

Radiotherapy is an alternative option of treatment if surgery is contraindicated or if negative surgical margins cannot be achieved with acceptable morbidity [15]. Other therapies for recurrent disease include locoregional interventions such as arterial embolization [16] or antiangiogenic therapy with interferon alpha [14], as well as systemic therapy including denosumab [17, 18]. Denosumab is a fully humanized monoclonal antibody that inhibits the receptor activator of nuclear factor κB ligand (RANKL). It has a significant role in clinical practice for treating unsalvageable GCTs, with a favorable efficacy and safety profile. However, it is limited by its lack of long-term evaluation and potential side effects, such as hypocalcemia, adverse impact on bone density, and osteonecrosis of the jaw. Neoadjuvant denosumab could promote en bloc resection, but local disease recurrence is not improved by neoadjuvant denosumab [19]. Traub et al. conducted a prospective, nonrandomized study of patients who received denosumab for 6–11 months before surgery and reported a local recurrence rate comparable to that reported in other studies without denosumab treatment [20].

## Conclusion

Giant cell tumor of the sternum is a rare diagnosis. Surgical resection with negative margins is the ideal management. To avoid defects or instability of the chest wall, reconstruction of the chest wall with neosternum should be considered.

## Data Availability

The data of this article are included within the article.
